# Early variations in *plasmodium falciparum *dynamics in Nigerian children after treatment with two artemisinin-based combinations: implications on delayed parasite clearance

**DOI:** 10.1186/1475-2875-9-335

**Published:** 2010-11-22

**Authors:** Obaro S Michael, Grace O Gbotosho, Onikepe A Folarin, Titilope Okuboyejo, Akintunde Sowunmi, Ayoade MJ Oduola, Christian T Happi

**Affiliations:** 1 Department of Pharmacology and Therapeutics, University of Ibadan, Ibadan, Nigeria; 2 Malaria Research Laboratories and Clinics, Institute of Advanced Medical Research and Training (IMRAT), College of medicine, University of Ibadan, Ibadan, Nigeria; 3 Special Programme for Research and Training in Tropical Diseases (WHO/TDR), Geneva, Switzerland

## Abstract

**Background:**

Combination treatments, preferably containing an artemisinin derivative, are recommended to improve efficacy and prevent *Plasmodium falciparum *drug resistance. Artemether-lumefantrine (AL) and artesunate-amodiaquine (AA) are efficacious regimens that have been widely adopted in sub-Saharan Africa. However, most study designs ignore the effects of these regimens on peripheral parasitaemia in the first 24 hours of therapy. The study protocol was designed to evaluate more closely the early effects and the standard measures of efficacies of these two regimens.

**Methods:**

In an open label, randomized controlled clinical trial, children aged 12 months to 132 months were randomized to receive AL (5-14 kg, one tablet; 15-24 kg, two tablets and 25-34 kg, three tablets twice daily) or artesunate (4 mg/kg daily) plus amodiaquine (10 mg/kg daily) for three days. Peripheral blood smears were made hourly in the first 4 hours, 8 h, 16 h, 24 h, and daily on days 2-7, and on days 7, 14, 21, 28, 35, and 42 for microscopic identification and quantification of *Plasmodium falciparum*.

**Results:**

A total of 193 children were randomized to receive either AL (97) or AA (96). In children that received both medications, early response of peripheral parasitaemia showed that 42% of children who received AL and 36.7% of those who received AA had an immediate rise in peripheral parasitaemia (0-4 h after treatment) followed by a rapid fall. The rise in parasitaemia was significant and seems to suggest a mobilization of asexual parasites from the deep tissues to the periphery. Days 3, 7, 14, 28, and 42 cure rates in the per protocol (PP) population were > 90% in both groups of children. Both drug combinations were well tolerated with minimal side effects.

**Conclusion:**

The study showed the high efficacy of AL and AA in Nigerian children. In addition the study demonstrated the mobilisation of asexual parasites from the deep to the periphery in the early hours of commencing ACT treatment in a subset of patients in both study groups. It is unclear whether the early parasite dynamics discovered in this study play any role in the development of drug resistance and thus it is important to further evaluate this discovery. It may be useful for studies investigating delay in parasite clearance of artemisinin derivatives as a way of monitoring the development of resistance to artemisinin to assess the early effects of the drugs on the parasites.

## Background

The World Health Organization (WHO) recommends artemisinin-based combination therapy (ACT) for the treatment of malaria in countries experiencing resistance to anti-malarial drug monotherapy [1]. Artemisinin derivatives are noted for rapid reduction of parasite biomass [2]. This class of drug have very short half-lives and their use alone in monotherapy is associated with a high percentage of treatment failure. Artemisinins are conventionally used in combination with slower acting anti-malarial drugs. The rationale for combination therapy is to improve the cure rate of infections responding inadequately to mono-therapy and possibly to prevent or delay the emergence of resistance to other drugs [1]. Studies have confirmed that the advantages of combining artemisinins with partner drugs far exceed the disadvantages [3]. This has led to their widespread use as first line therapies worldwide.

The guidelines for the comparative studies of anti-malarial therapies have largely remained unchanged since the time of widespread use of chloroquine and other monotherapies. The standard endpoints of efficacy studies remain the evaluation of events around the following days of therapy; days 3, 7, 14, 28, and more recently day 42. Most studies on ACT using these standard endpoints have consistently reported similarities in efficacies of ACT with cure rates above 90% [4-10]. This is not surprising considering the fact that the artemisinins, the fastest anti-malarials, are components of both arms of comparative studies. In addition, the drugs kill parasites more rapidly than conventional anti-malarials, and are active against both the sexual and asexual stages of the parasite cycle. Artemisinin fever clearance time is shortened to 32 hours as compared with 2-3 days with older agents [11]. Using current protocols of evaluation of efficacy, subtle differences between different forms of ACT may be masked on account of the rapidity of anti-malarial effects of the Artemisinin component. In addition these masked differences may be involved in eventual reduction of susceptibility of parasites to ACT, as already being reported by some studies [12-14]. The need to evaluate the precise estimate of the risk of treatment failure with ACT using several methods of analysis has been attempted by some authors [15,16]. It has become important that more sensitive and robust methods of assessing the *in vivo *efficacies of ACT be evolved in view of the presence of artemisinins in both arms of studies evaluating ACT.

In this study, the comparative efficacy and safety of two different forms of ACT [artesunate-amodiaquine (AA) and artemether-lumefanthrine (AL)] was evaluated using a modified protocol to evaluate two time points of efficacy; early effects (≤ 72 hours of therapy) and late effects (> 72 h to 42^nd ^day of therapy). The early and late efficacy of these two different forms of ACT on peripheral asexual falciparum parasitaemia was assessed.

## Methods

### Study site, design and patients

The study was a parallel, open label, randomized controlled clinical trial conducted at the malaria clinic of the University College Hospital, Ibadan, Nigeria. Ibadan is located in the rain forest belt in south-western Nigeria. Malaria transmission is intense and occurs all year round in the study area. Children aged 12 months to 132 months were enrolled in the study in accordance with the following inclusion criteria: signs and symptoms compatible with acute uncomplicated malaria, axillary temperature > 37.4°C, microscopically confirmed asexual forms of *P. falciparum *with parasite density of > 2,000 parasite/μL, willingness to comply with protocol, and parental guardian written informed consent. Children with severe malnutrition, presence of mixed infection, signs of severe and complicated malaria or other febrile illnesses and history of hypersensitivity to any of the study drugs were excluded from the study.

### Ethical issues

The study was conducted in accordance to Good Clinical Practice and the Helsinki declaration. Ethical clearance was obtained from the University of Ibadan/University College Hospital Joint Institutional Review Committee. A written or witnessed verbal informed consent was obtained from the parent or guardian of each child before any study-related procedure was carried out.

### Treatment allocation and case management

At enrolment, children who satisfied the enrolment criteria were examined thoroughly and weighed. Thick and thin blood films were prepared from a finger prick under aseptic conditions and blood spots collected on filter paper for molecular analysis at enrolment.

Enrolled children were allocated to one of two treatment groups according to a pre-generated randomization table. Randomization was done using a computer generated random sequence. These sequences were concealed in envelopes prior to commencement of the study. However, the study was not blinded. The study physician opened the drug envelopes only after the children were allocated according to the computer generated drug sequence. Children randomized to group 1 received AL twice daily for three days. AL was given according to the weight of enrolled children. Children weighing 5 to < 15 kg received one tablet, those weighing 15 to < 25 kg received two tablets while those weighing 25 to < 35 kg received three tablets twice daily for 3 days. Each tablet of AL contained 20 mg artemether and 120 mg lumefantrine. Patients randomized to group 2 received AA. Artesunate was administered at a dose of 4 mg/kg body weight once daily for three days and amodiaquine at a dose of 10 mg amodiaquine base/kg body weight once daily for three days. The tablet formulations of both drugs were crushed and dissolved in milk (AL) or water (AA) before administration. Study drugs were administered supervised by a nurse or a physician. The enrolled patients were admitted to hospital ward for three days for observation of drug administration and clinical states. After administration of drugs, patients were observed for 30 min and the dose was re-administered if the patient vomited within the period. Febrile children with axillary temperature ≥38°C were exposed, tepid-sponged and given oral paracetamol. Children were withdrawn for the following reasons: withdrawal of consent, protocol violation, repeated vomiting or loss to follow-up.

### Patient follow-up

Study participants were followed up daily on days 0-7 and on days 14, 21, 28, 35 and 42. At each visit children were physically examined and their vital signs recorded. In addition, thick blood films were prepared from finger prick blood samples. Thick blood films were prepared hourly for the first 4 hours, at 8, 16 and 24 hours (day 0) following therapy. Subsequently thick films were made daily on days 1-7, 14, 21, 28, 35 and 42. These were stained with 10% fresh Giemsa stain and examined for the presence of asexual forms of *P. falciparum *under a light microscope at 1,000× magnification. Blood spots were also collected on filter paper for molecular analysis at entry and daily for the first 7 days, on days 14, 21, 28, 35 and 42.

Children who were not brought to the clinic on scheduled days were visited at home for the follow-up procedures. Each child and the parent or guardian was questioned about symptoms observed following commencement of therapy. Children who failed treatment were re-treated with artesunate-mefloquine.

### Evaluation of efficacy

Efficacy was comparatively evaluated using two time points. Early drug effects were classified as effects occurring between 0 h and < 72 h of treatment. Late effects were those occurring between > 72 h and day 42 post-treatment.

Early treatment failure (ETF), defined as development of danger signs of malaria or severe malaria on post-treatment days 1, 2 or 3, in the presence of parasitaemia, parasitaemia on day 2 higher than day 0 count irrespective of axillary temperature, parasitaemia on day 3 which is > 25% of count on day 0 and parasitaemia on day 3 with axillary temperature 37.5°C [17].

Late clinical failure (LCF) was defined as development of danger signs or severe malaria after day 3 in the presence of parasitaemia without previously meeting any of the criteria of ETF and presence of parasitaemia and axillary temperature of 37.5°C on any day from day 4 to day 42, without previously meeting any of the criteria of ETF.

Late parasitological failure (LPF) was defined as presence of parasitaemia on any day from day 7 to day 42 and axillary temperature of < 37.5°C without previously meeting any of the criteria of ETF or LCF.

Adequate clinical and parasitological response (ACPR) was defined as absence of parasitaemia on day 42 irrespective of axillary temperature without previously meeting any of the ETF, LCF or LPF.

Discrimination between recrudescence and re-infections in treatment failures was done using molecular analysis [18,19]. Isolates from each *P. falciparum *infection in the study were characterized on the basis of the fragment size of alleles of msp-2 after amplification by PCR. A recrudescent infection was defined as the occurrence of the same or a subset of the alleles at each of the families (FC27 or IC1/3D7) of msp-2 in the primary and post-treatment samples. A lack of allelic identity in the two families of msp-2 in matched primary and post-treatment samples indicated a newly acquired infection. Infections were defined as polyclonal if parasites in matched primary and post-treatment samples from the same patient showed more than one allele of FC27 or IC1/3D7 families of msp-2. If an isolate had one allele at each of the families, the clone number was taken to be one.

### Assessment of drug related adverse events

An adverse event (AE) was defined as signs, symptoms or abnormal laboratory finding not present at enrolment, but occurring during follow-up, or being present at day 0 and becoming worse during follow-up despite clearance of parasitaemia. All AEs were monitored and recorded. Assessment was by asking about the progress of presenting symptoms and new symptoms noticed during follow-up, physical examination, and or laboratory evaluation.

### Data analysis

Data collected were recorded in case record forms and entered into Epi-info version 6.04 database for analysis. SPSS version 16 was also used for analysis. Efficacy analysis of the data was done for the intention to treat (ITT) and per protocol (PP) populations. All children enrolled into the study were considered ITT. Patient who completed the study without violating the study protocol were considered as PP population. Parasite densities are reported as geometric mean parasite densities. The means and standard deviations (± SD) of normally distributed data were compared using Student's t-test and analysis of variance (ANOVA). Proportions were compared by chi-square. Numerical values are given as means ± SD, p-values < 0.05 was taken as statistically significant.

## Results

**Figure 1:**
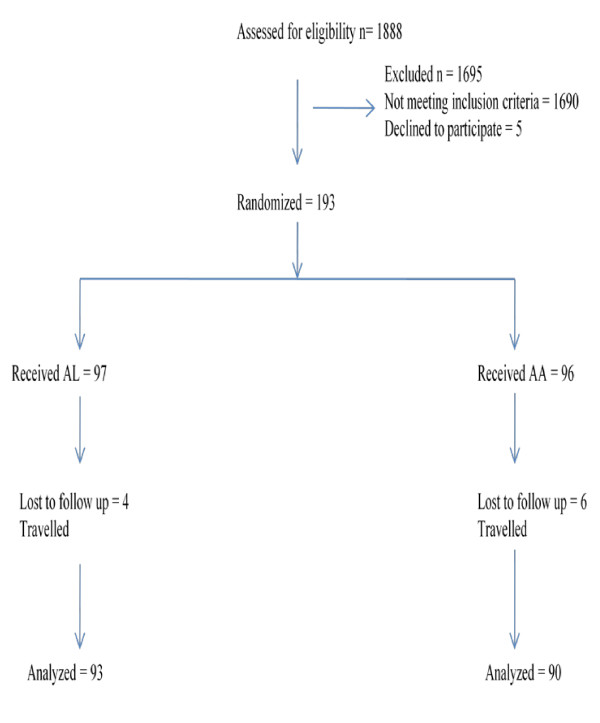
**Trial Profile of Nigerian children enrolled in a AA and AL efficacy study conducted at the Malaria Clinic, Malaria Research Laboratories, College of Medicine, University of Ibadan, Nigeria**.

A total of 1,888 children were screened for the study and 193 consented after meeting the inclusion criteria. Ninety-seven (97) children were randomized to receive AL and 96 were randomized to receive AA. Ten (10) of the children enrolled (5.2%) were lost to follow-up: four patients from AL group and six (6) from AA group (Figure 1).

The baseline characteristics of study participants in the two groups were similar. The three most common presenting complaints were fever (100%), vomiting (51%) and abdominal pain (35%). Ninety-three of 97 (95.9%) patients treated with AL completed the study while 90/96 (93.7%) of those that receive AA completed the study (per protocol population) (Table 1).

**Table 1:** Pre-enrolment characteristics of 183 children involved in the study (per protocol population).

Variable	AL (N = 93)	AA (N = 90)
Male:female	45:48	43:47
Age (yr)	6.3 ± 2.5	6.8 ± 2.6
95% CI	5.95 - 7.38	5.89 - 7.38
Duration of illness (d)	2.7 ± 1.5	2.5 ± 1.1
95% CI	2.32 - 2.16	2.21 - 2.87
Weight (kg)	18.2 ± 5.3	18.9 ± 4.6
95% CI	17.52 - 20.52	17.7 - 20.26
Temperature°C	38.4 ± 1.2	38.2 ± 1.2
95% CI	38.05 - 38.77	37.96 - 38.64
Hematocrit (%)	32.6 ± 3.4	32.5 ± 3.9
95% CI	31.88 - 33.80	31.28 - 33.60
GMPD (/μl of blood)	78195	69288
Range	2837 - 1105263	2791 - 1125000

AL = artemether-lumefanthrine; AA = artesunate-amodiaquine

GMPD = geometric mean parasite density; SD = standard deviation

d = days; *values are given as mean ± SD.

### Early Efficacy evaluation

Responses to therapy were good and prompt in all study participants. Mean fever clearance times (FCT) for AL and AA were 29.9 ± 18.4 vs. 28.9 ± 12.7 hours, respectively (p = 0.630). The 24 and 48-h parasite reduction ratios (PRR) were 11 × 10^3^, 78 × 10^3^, and 14 × 10^3^, 69 × 10^3 ^for AL and AA (p = 0.562, 0.534), respectively, while mean parasite clearance times (PCT) were 28.6 ± 18.4 hours AL and 24.0 ± 15.4 hours for AL and AA respectively (p = 0.067). Cure rates by day 3 in both groups were 100% (Table 2). The overall patterns of parasite reduction in children in the two groups were similar.

**Table 2:** Therapeutic responses of the enrolled children after treatment with AL or AA.

Treatment groups			
	**AL**	**AA**	**p-value**

P↑ %	42	36.7	0.563
PRR 24 h	11 × 10^3^	14 × 10^3^	0.279
FCT (hours)	29.9 ± 18.4	28.9 ± 12.7	0.630
95% CI	26.83 - 32.82	25.81 - 31.93	
PCT (hours)	28.6 ± 18.4	24.0 ± 15.4	0.067
95% CI	25.23 - 33.79	20.71 - 28.13	
ETF	0	0	1.000
LPF	7.5%	7.8%	0.830
ACPR	92.5%	92.2%	0.830
PCR-corrected cure rates	93.5%	93.3%	0.955

P↑ proportion with increase in peripheral blood parasitaemia in the first few hours of therapy

PRR = parasite reduction ratio at 24 hours; FCT = fever clearance time;

PCT = parasite clearance time; ETF = early treatment failure; LPF = late treatment failure

ACPR = adequate clinical and parasitological response; Values are reported as mean ± SD or as proportions

### Effects on early parasite dynamics

**Figure 2:**
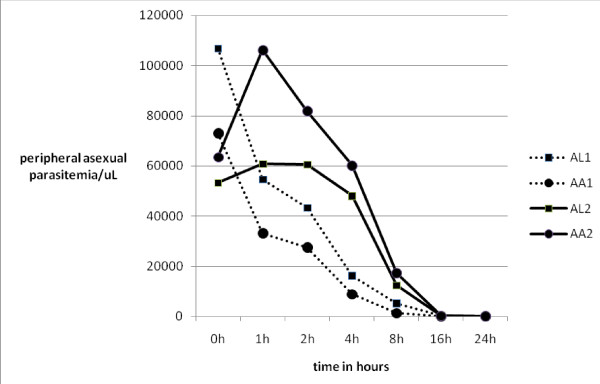
**Mean peripheral parasitaemia in the first 24 h in children with increased parasitaemia during the first hours of treatment and in those who showed no increase in peripheral asexual parasitaemia**. AL1: children who did not have initial increase in peripheral parasitaemia after treatment with artemether-lumefanthrine AL2: children who had an increase in peripheral parasitaemia in the first few hours after commencing treatment with artemether-lumefanthrine AA1: children who did not have initial increase in peripheral parasitaemia after treatment with Artesunate-amodiaquine AA2: children who had an increase in peripheral parasitaemia in the first few hours after commencing treatment with Artesunate-amodiaquine.

Analysis of drug effects on hourly changes in peripheral asexual parasitaemia in the first 24 hours following treatment showed that there were two sub-groups; those in whom parasitaemia increased in the first 4 hours after the first dose of the medications and those that did not show any increase in peripheral parasitaemia (Figure 2). Pre-enrolment characteristics of these two sub-groups were similar (Table 3). Forty-two percent (42%) of the patients who received AL had an initial elevation of peripheral parasitaemia, while the remaining did not. In those that received AA 36.7% showed an initial increase in peripheral parasitaemia. This proportion of children that showed initial increase in parasitaemia in the two study groups was similar (42% vs. 36.7%, p-value 0.377). Parasitaemia in patients that showed increase in peripheral asexual parasitaemia peaked at one hour after the first dose of AL or AA. Peripheral asexual parasitaemia in children who did not show initial increase and those that showed increase were the same at all time points except at 4 and 8 hours. Geometric means of parasitaemia at 4 and 8 hours after first dose of the study drugs were 10579 parasites/μL vs 55,809 parasites/μL (p = 0.011) and 2,297 parasites/μL vs. 15,409 parasites/μL (p = 0.002) for sub-groups that showed no increase and increase respectively.

**Table 3:** Baseline parameters (at enrolment) between the two sub-groups of patients that showed different peripheral asexual parasitaemia in the immediate hours following administration of the first dose of treatment medications (all patient analysis, intention to treat population).

Parameters	Sub-group with no increase in peripheral asexual parasitaemia following onset of therapy	Sub-group with initial increase in peripheral asexual parasitaemia following onset of therapy
Number (%) N = 193	118 (60.9%)	75 (39.1%)
Sex ratio M:F	61:56	34:42
Mean age (years) ± SD	6.6 ± 2.5	6.6 ± 2.7
95% CI	4.71 - 7.72	5.01 - 8.72
Mean weight (kg) ± SD	18.6 ± 5.1	18.7 ± 5.0
95% CI	15.98 - 23.30	14.99 - 20.28
Mean duration of fever (days) ± SD	2.7 ± 1.5	2.7 ± 1.1
95% CI	1.98 - 3.87	1.74 - 3.89
Mean temperature (°C) ± SD	38.3 ± 1.2	38.3 ± 1.2
95% CI	37.78 - 39.13	38.18 - 39.74
Mean hematocrit (%) ± SD	33.1 ± 3.6	32.1 ± 3.7
95% CI	29.90-33.53	28.89 - 33.29
Geometric Mean of 0 hour parasitaemia	82870	60437
Range	2837 - 1105263	2657 - 1125000

CI = Confidence Interval

**Figure 3:**
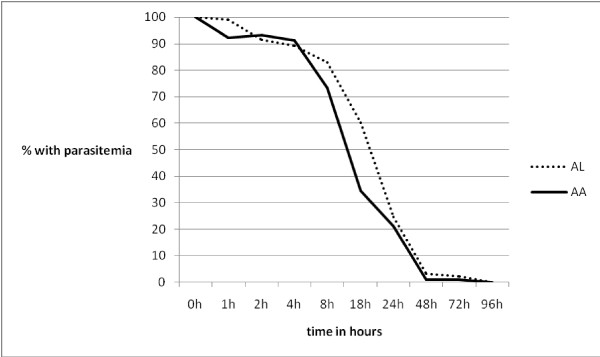
**Proportion of children with peripheral asexual parasitaemia plotted against time (hours)**.

In the AL and AA groups, peripheral asexual parasite changes in the first 24 hours and at others time points of the study were similar between the two sub-groups described above. Geometric means of parasitaemia at 0 hour and 1 hour in the two subgroups of AL and AA in those that showed no increase in parasitaemia in the first hour following therapy were 99,028 parasites/μL and 50,075 parasites/μL (for AL), and 72,927 parasites/μL and 33,167 parasites/μL (for AA) respectively (p-values 0.133 and 0.302). Geometric means of parasitaemia at 0 hour and 1 hour in the two subgroups of AL and AA in those that showed increase in parasitaemia in the first hour following therapy were 53,001 parasites/μL and 60,744 parasites/μL (for AL) and 63,424 parasites/μL and 106,185 parasites/μL (for AA) [p-values 0.698 and 0.248]. PRR in the first 24 hours and 48 hours were similar in both groups (Figure 3).

### Late efficacy evaluation

Intention to treat (ITT) population: Day 14, 28, and 42 cure rates for AL and AA were 100%, 97.9%, 92.8%, and 100%, 96.9%, 93.8% respectively. PCR corrected cure rates on day 28, 42 for AL and AA were 97.9%, 94.8%, and 97.9%, 93.8% respectively. Per-protocol (PP) population: Day 14, 28, and 42 cure rates for AL and AA were 100%, 96.8%, 92.5%, and 100%, 97.8%, 92.2% respectively. PCR corrected cure rates on day 28, 42 for AL and AA were 97.8%, 93.5%, and 97.8%, 93.3% respectively (Table 2). All rates were similar between the two drug groups.

### Safety evaluation

The study drugs were well tolerated. All recorded adverse events were mild and transient. Mean resolution time for all adverse drug events reported during the study was 2.86 ± 1.8 days. Twelve children in the study reported adverse drug events. These adverse drug events were reported within the first week of treatment. Common adverse events reported were abdominal pain, vomiting, weakness, and headache. A child who was treated with AL developed a transient generalized papular rash with itching. No child had therapy discontinued or modified on account of adverse drug event Table 4 is a summary of adverse drug events reported by the children during the study.

**Table 4:** Adverse drug events reported within the first week of the study.

	AL	AA
No. of children	4 (4.4%)	8 (8.3%)
Abdominal pain	2 (2.2%)	3 (3.1%)
Vomiting	0 (0%)	2 (2.1%)
Weakness	0 (0%)	2 (2.1%)
Headache	1 (1.1%)	1 (1.0%)
Skin rash with itching	1 (1.1%)	0 (0%)

AL artemether-lumefanthrine; AA artesunate-amodiaquine

## Discussion

Artemisinin based combination therapy (ACT) was formally introduced in Nigeria in 2005 following the progressive decline in efficacy of chloroquine and sulphadoxine-pyrimethamine. The two most widely adopted ACT regimens are AL and AA [20]. The results of this study showed that both combination therapies were safe and well tolerated. In addition, AA and AL rapidly eliminated asexual parasites and they retain their high efficacy in Nigerian children. Overall, the study showed a high efficacy of the two forms of ACT in Nigerian children; cure rate was above 90% for both groups of children. These results are consistent with the high cure rates known to be associated with ACT in other malaria endemic areas of Africa [21-23], although other reports suggest a marginally higher day 28 PCR-corrected cure rate for AL over AA [24]. Generally, studies from East Africa seem to suggest that AL has more clinical benefits than AA, while studies from West Africa show that AA tend to have a marginally better efficacy over AL [25].

The effects of the study drugs on asexual parasites immediately following treatment uncovered an unexpected finding. Two patterns of asexual parasite responses occurred; children that experienced an acute rise in peripheral parasitaemia hours after commencing treatment and those that did not. This effect was uncovered due to frequent blood sampling for malaria parasite evaluation in the first 24 hours of the study. Daily sampling, as recommended by the WHO, would have missed this important finding because these effects occurred within 24 hours of initiating treatment. Parasitaemia reached a peak at 1 hour in the sub-group of children that had an initial rise in peripheral parasitaemia after commencing therapy. Analysis of demographic and clinical parameters of children in these two sub-groups (Table 3) did not point to any factor that may explain this acute rise in parasitaemia after starting treatment, although pre-treatment parasite density was marginally lower in the sub-group of children that had an initial acute rise in peripheral parasitaemia at commencement of therapy compared to those that did not (72,927 parasites/μL vs 99,028 parasites/μL p-value 0.133). This marginal difference may point to the fact that children who had a rapid rise in asexual parasitaemia after commencing treatment might have had a larger load of sequestered parasites in the deep tissues. This acute mobilization of asexual parasites from the deep tissues to the periphery may be attributable to the action of ACT in enhancing the clearance of the parasites from the blood by exposing the parasites to the drug and driving them to the spleen where they are cleared from the blood (Happi and Sowunmi, personal communication). In addition, the mobilization of parasites from the deep tissues by ACT may be responsible for reduction in the incidence of development of severe and complicated malaria attributed to ACT [26]. Other explanations may be that a proportion of native *P falciparum *in the Nigerian population has a higher tendency to sequestration, or that a proportion of the children have a tendency to having more of sequestered parasites. These postulates remain to be fully explored.

Studies in the past have described increase in parasitaemia 6 to 12 hours after administration of parenteral anti-malarial drugs and have attributed this phenomenon of natural progression of sequestered parasites on account of a lag period between drug administration, absorption and onset of parasitocidal effects of the drugs [27]. The lag period allows the natural multiplication of parasites to continue until the drugs start acting. Silachamroon *et al *[28], in a retrospective study in 2001, reported that early rising parasitaemia after treatment with artemisinin derivatives was very common despite their ability to clear parasites from the blood. These authors stated that 25.8% of the patients with uncomplicated malaria and 41.3% of those that had complicated malaria encountered early rise in parasitaemia.

The clinical efficacy of artemisinin is known to be characterized by an almost immediate onset and rapid reduction of parasitaemia [29]. However, this study showed that before the rapid decline in parasitaemia on account of drug action, there is a brief interval of rapid rise in parasitaemia within the first four hours of starting ACT treatment in a subgroup of children followed by a rapid clearance within 24 hours. Although the reasons behind the initial rise in parasitaemia in some patients as shown in this study remain unclear, Silachamroon and colleagues [28] have reported previously that early rising parasitaemia in falciparum malaria treated with artemisinin derivatives is not associated with eventual treatment outcome.

Based on the observations of potential mobilization of parasites from the tissues to the periphery in this study, it is possible that AA and AL may have a property of rapidly releasing parasites from sequestration sites in the deep tissues. It is not clear if the early parasite dynamics discovered in this study play any role in the development of drug resistance and thus it is important to further evaluate this finding. Studies investigating delay in parasite clearance of artemisinin derivatives as a way of monitoring the development of resistance to artemisinin should also assesses the early effects of the drugs on the parasites.

## Conclusions

The two forms of ACT studied, AL and AA, had similar efficacies and effects on peripheral asexual *Plasmodium falciparum *parasitaemia in the treatment of acute uncomplicated malaria in Nigerian children. Therapeutic responses were rapid, and efficacy at day 42 was above 90% for both combinations. Both drugs were well tolerated with minimal side effects.

The study also revealed that asexual parasite dynamics in the early hours of initiating therapy showed two patterns; those who had an early rise in peripheral parasitaemia immediately after treatment and those who did not. The significance of this finding remains to be further explored and explained.

## Competing interests

The authors declare that they have no competing interests.

## Authors' contributions

HCT, GOG, AS and AMJO, conceived and designed of the study. OSM, TO, HCT, GOG, OAF and AS participated in patients enrolment, samples collection and data analysis. OSM and HCT drafted the manuscript. All the authors read, made suggestions and approved the manuscripts that were submitted for review and publication.
